# Genomic history of human monkey pox infections in the Central African Republic between 2001 and 2018

**DOI:** 10.1038/s41598-021-92315-8

**Published:** 2021-06-22

**Authors:** Nicolas Berthet, Stéphane Descorps-Declère, Camille Besombes, Manon Curaudeau, Andriniaina Andy Nkili Meyong, Benjamin Selekon, Ingrid Labouba, Ella Cyrielle Gonofio, Rita Sem Ouilibona, Huguette Dorine Simo Tchetgna, Maxence Feher, Arnaud Fontanet, Mirdad Kazanji, Jean-Claude Manuguerra, Alexandre Hassanin, Antoine Gessain, Emmanuel Nakoune

**Affiliations:** 1 The Center for Microbes, Development and Health, CAS Key Laboratory of Molecular Virology and Immunology, Institut Pasteur of Shanghai-Chinese Academy of Sciences, Discovery and Molecular Characterization of Pathogens, No. 320 Yueyang Road, XuHui District, Shanghai, 200031 China; 2 Institut Pasteur, Unité Environnement et Risque Infectieux, Cellule d’Intervention Biologique d’Urgence, Paris, France; 3 Centre International de Recherches Médicales de Franceville (CIRMF), Franceville, Gabon; 4 Institut Pasteur, Centre of Bioinformatics, Biostatistics and Integrative Biology (C3BI), Paris, France; 5 Institut Pasteur, Emerging Diseases Epidemiology Unit, Paris, France; 6 Institut de Systématique, Évolution, Biodiversité (ISYEB), Sorbonne Université, MNHN, CNRS, EPHE, UA, Paris, France; 7 Institut Pasteur de Bangui, Bangui, Central African Republic; 8 Unité Pasteur-CNAM Risques Infectieux et Emergents (PACRI), Conservatoire National des Arts et Métiers, Paris, France; 9 Institut Pasteur, Unité d’Epidémiologie et Physiopathologie des Virus Oncogènes, Département de Virologie, Paris, France; 10 Centre National de Recherche Scientifique (CNRS) UMR3569, Paris, France

**Keywords:** Phylogenetics, Pox virus, Viral evolution

## Abstract

Monkeypox is an emerging infectious disease, which has a clinical presentation similar to smallpox. In the two past decades, Central Africa has seen an increase in the frequency of cases, with many monkeypox virus (MPXV) isolates detected in the Democratic Republic of Congo (DRC) and the Central African Republic (CAR). To date, no complete MPXV viral genome has been published from the human cases identified in the CAR. The objective of this study was to sequence the full genome of 10 MPXV isolates collected during the CAR epidemics between 2001 and 2018 in order to determine their phylogenetic relationships among MPXV lineages previously described in Central Africa and West Africa. Our phylogenetic results indicate that the 10 CAR isolates belong to three lineages closely related to those found in DRC. The phylogenetic pattern shows that all of them emerged in the rainforest block of the Congo Basin. Since most human index cases in CAR occurred at the northern edge of western and eastern rainforests, transmissions from wild animals living in the rainforest is the most probable hypothesis. In addition, molecular dating estimates suggest that periods of intense political instability resulting in population movements within the country often associated also with increased poverty may have led to more frequent contact with host wild animals. The CAR socio-economic situation, armed conflicts and ecological disturbances will likely incite populations to interact more and more with wild animals and thus increase the risk of zoonotic spillover.

## Introduction

Monkeypox, an emerging and neglected infectious disease, is caused by the monkeypox virus (MPXV), which belongs to the genus *Orthopoxvirus* in the family *Poxviridae*^1^. The disease has a clinical presentation similar to smallpox, with additional symptoms including adenopathy and maculopapular rash, especially on the palms of the hands and the soles of the feet^2^. Although there is no specific treatment or vaccine for monkeypox, cross-immunity with smallpox vaccination may offer some protection in human populations^3^. Nonetheless, the monkeypox lethality rate varies from 1 to 10%^4^. Transmission of MPXV in humans occurs either through direct contact with infected animals or through contact with body fluids or respiratory droplets of an infected person, resulting in secondary transmission. Although monkeypox is a zoonotic disease, the animal reservoir has not yet been identified. MPXV has been isolated twice from wild animals: in 1985 from a symptomatic Thomas’s rope squirrel (*Funisciurus anerythrus*) caught in the Democratic Republic of the Congo (DRC)^5^ and in 2012 from a sooty mangabey (*Cercocebus atys*) in Ivory Coast^6^. More recently, MPXV was sequenced from the feces of chimpanzees in Ivory Coast^7^. Serological studies have suggested, however, that several rodent species are potential reservoirs^8^.

In the past two decades, Congo Basin MPXVs have primarily affected two Central African countries, the DRC and the Central African Republic (CAR), at a growing case rate ^3,9–11^. In 2018, monkeypox human cases were also reported in western Cameroon ^12^, near the border with the Nigeria. The sequence of the ATI gene (639 bp) was obtained from one of these cases. Given the close phylogenetic relationship identified between the Cameroonian (for the 2018 strain) and Nigerian isolates, the close geographical proximity of the affected regions and the absence of human cases in Cameroon since 1989, the origin of this isolate is likely an import from Nigeria (West Africa). However, as also suggested by the authors, the hypothesis of a natural circulation within Cameroon cannot be completely ruled out^12^. Monkeypox cases caused by the West African clade of MPXV have recently been identified outside of the African continent^13,14^. Three human cases were diagnosed in the United Kingdom (UK), the first of which involved a person traveling through Paris from Nigeria, and the second resulting in a nosocomial transmission to a health worker in the UK^15^. Additional cases have recently been imported into Israel and Singapore from Nigeria^16,17^.

**Figure 1:**
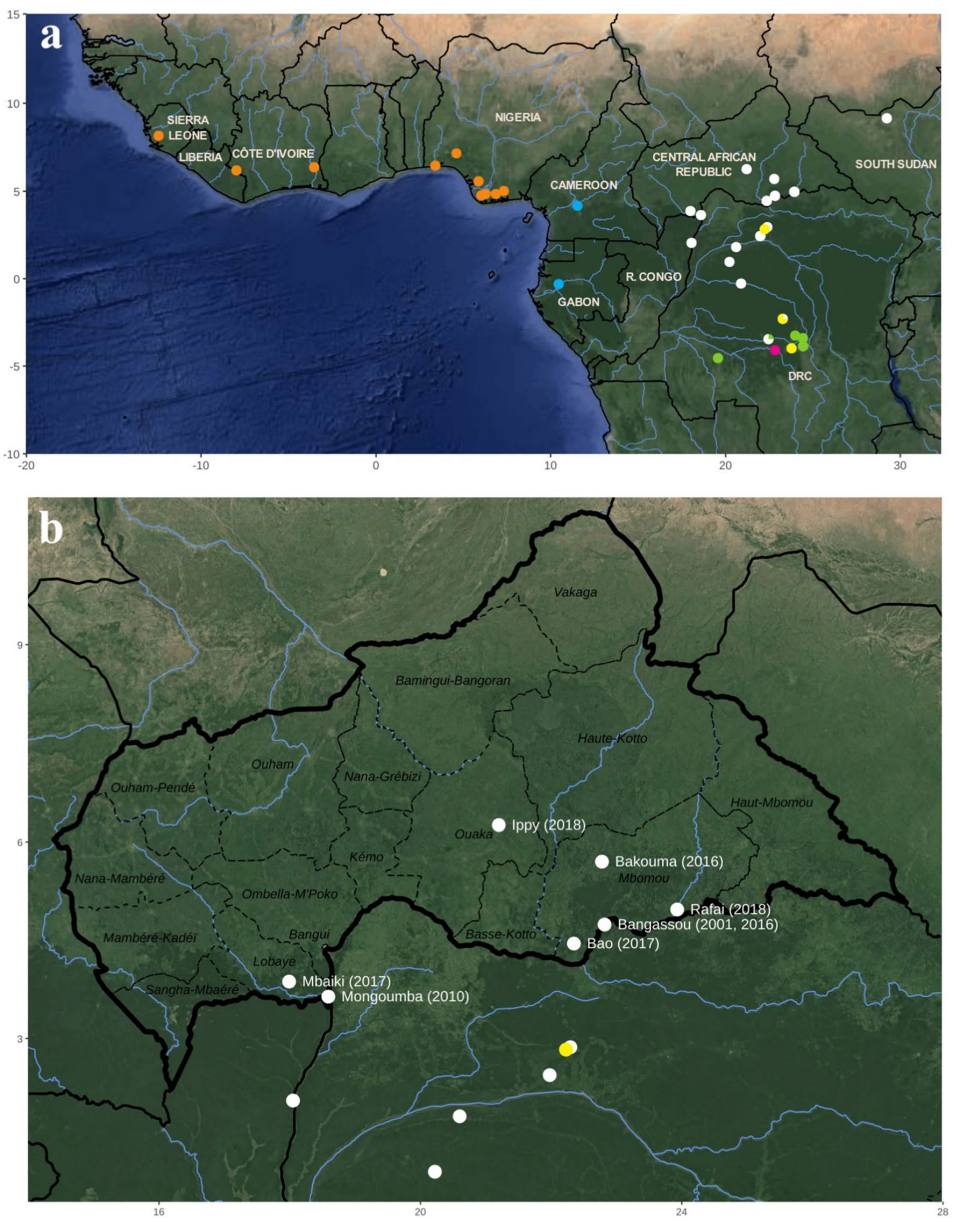
Geographic origin of the 56 monkeypox virus (MPXV) isolates analysed in this study (**a**). The 10 MPXV isolates collected in the CAR and specially sequenced for this study are indicated in the map of (**b**). The localities of reporting and years of these MPXV cases are indicated in white. As detailed in Fig. 2, the MPXV isolates belong to the West African clade (orange) or to the Central African groups I (blue), II (white), III (yellow), IV (green) and V (pink). The map was created with ggmap v3.0.0 under R 4.0.456. Maps Data: Google, 2021 NASA/TerraMetrics.

Many MPXV genomes have been sequenced from previous outbreaks in the DRC and Nigeria, but no genomic data is currently available for MPXV detected over the past two decades in the CAR. In Central Africa, the Congo Basin is a large block of tropical rainforests covering, from West to East, Equatorial Guinea, Gabon, southern Cameroon, the southern and northern parts the Republic of the Congo (R. Congo), the northern half of the DRC, and several southern regions of the CAR (Sangha-Mbaéré, southern Mambéré-Kadéï, southern Lobaye, southern Mbomou; see details in Fig. 1). By contrast, northern regions of the CAR are mainly composed of savannah habitat-types. Obtaining genomic data from viruses isolated between 2001 and 2018 in the CAR can be therefore very interesting to better understand how outbreaks emerged at the ecotone between rainforests and savannahs.

**Figure 2:**
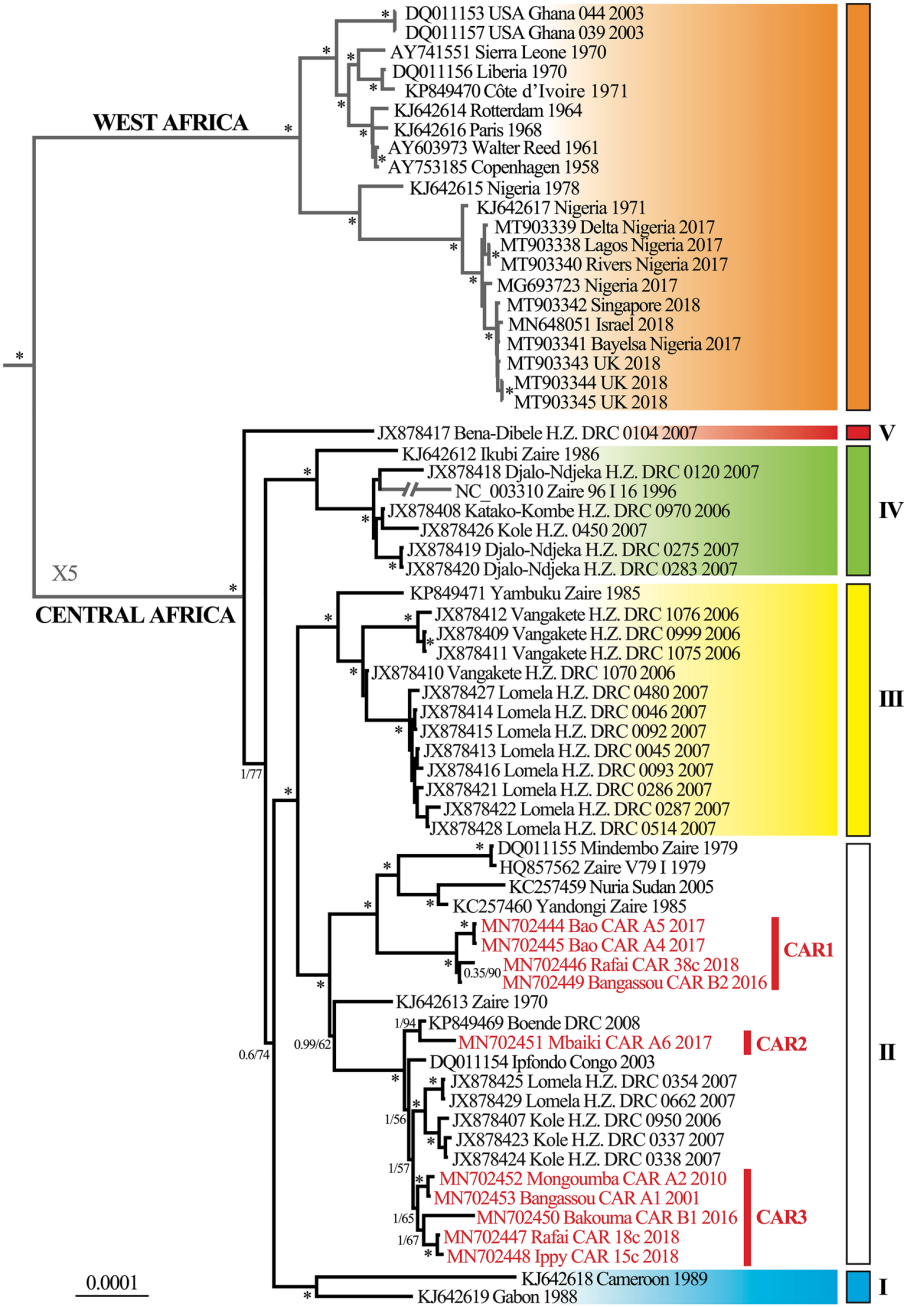
Phylogeny of monkeypox viruses (MPXV) based on complete genomes. The Bayesian tree was reconstructed using the GTR+G model. The two sequences used as outgroup are not shown. For each MPXV sequence, we indicated the accession number in GenBank, the geographic locality, the country, the code, and the year. The 10 MPXV genomes specially sequenced for this study are written in red. Branches with an asterisk were supported by maximal Bayesian posterior probability (PP = 1) and ML bootstrap proportions ≥ 95. The main clades were highlighted with different colours: West African clade (orange), Central African group I (blue), II (white), III (yellow), IV (green) and V (pink). For convenience, the size of longest branches shown in grey was reduced by five.

## Materials and methods

### Organization of the field mission for investigations of outbreaks or cases

All suspected cases of monkeypox in the CAR were investigated according to a well-established procedure validated by both the CAR Ministry of Health and the World Health Organization (WHO)^11^. The local health authorities used focal points present in all regions to be able to rapidly detect all suspected cases. Samples (blood, scabs and pus) were collected by Institut Pasteur Bangui (IPB) staff, and stored in a refrigerated compartment during the transportation to the IPB. The tools used for the notification of suspected cases, the collection of biological samples (scabs and/or pus) to investigate the etiological cause, and the registration of epidemiological data have been standardized and validated by the CAR authorities. As soon as a case was reported, a team was commissioned to go into the outbreak area to carry out field investigations and interview officials from the local health care teams. Outbreak investigations consisted of the identification of human monkeypox cases and their contacts to establish the chain of transmission, including suspicious human contacts with wild animals. Moreover, they also looked at patient consultation records, health records, and hospitalization forms to supplement the epidemiological data. The term micro-outbreak that we used in our article corresponds to us to outbreak with less that ten human cases.

### Isolation of MPXV, DNA extraction and molecular assays

Two MPXV isolates (A1 and A2; Table 1) were isolated and amplified after one passage of intracranial inoculation in the brains of eight neonatal mice (24–72 h old) using serum, pus or scab homogenates from patients, as previously described^19^. The mice were monitored daily for a week until mortality or morbidity was observed in at least one mouse. Then, the brains of the dead mice were harvested and suspended in a 1 × PBS solution (pH = 7.4). For all samples, whether harvested from mouse brains, pus or scabs (Table 1), DNA was extracted using the QIAamp viral DNA Mini kit, according to the manufacturer’s instructions. Extracted DNA was stored at − 20 °C until use in molecular investigations. MPXV was detected using quantitative and conventional polymerase chain reaction (PCR) on DNA extracted from clinical samples or after isolation, as previously described^20,21^.

**Table 1:** Metadata on monkeypox virus (MPXV) case samples.

Sample ID*	Prefecture**	City**	Sample collection date (dd/mm/yy)	Age (years)	Sex***	Suspected animal host or contact with humans****	Accession number	References
A1	Mbomou	Bangassou	11/08/01	14	M	Monkey	MN702453	^4^
A2	Lobaye	Mongoumba	10/08/10	15	M	Cetartiodactyla (*Cephalophus sylvacultor*)	MN702452	^10^
B1	Mbomou	Bakouma	01/01/16	37	F	Unknown	MN702450	^11^
B2	Mbomou	Bangassou	02/01/16	33	F	Rodent (*Thryonomys*)	MN702449	
A4	Mbomou	Bao	06/02/17	12	F	Unknown	MN702445	^22–24^
A5	Mbomou	Bao	06/02/17	06	M	Unknown	MN702444	
A6	Lobaye	Mbaiki	14/04/17	15	M	Unknown	MN702451	^22–24^
15c	Ouaka	Ippy	07/03/18	29	M	Contact with a fish with eruptive rash	MN702448	^25^
18c	Mbomou	Rafai	20/03/18	26	F	Direct contact with infected human (15c)	MN702447	
38c	Mbomou	Rafai	13/04/18	31	M	Unknown	MN702446	

*Sample ID assigned during sample collection from patients with MPXV infections in the CAR on the date indicated in the ‘Sample collection date’ column.

**Prefecture/City of the observed MPXV disease case, corresponding to sample collection location.

***M: Male, F: Female.

**** Determined from field investigations in the populations involved.

### Double-capture library preparation and sequencing

The libraries were prepared using the SureSelect XT library preparation kit for the Illumina sequencing kit. The SureSelect biotinylated probes for targeted enrichment were custom designed and are specific to a panel of known complete MPXV genomes (GenBank accession number: KJ642618, KJ642619, KJ642613, DQ011154, JX878423, DQ011155, KC257459, KC257460, JX878409 and NC_003310). DNA was quantified using a Qubit 2.0 fluorometer (Invitrogen) and at least 200 ng of DNA (suspended in 50 μl) was fragmented using a Covaris M220 ultrasonicator (Covaris, Woburn, MA, USA). This fragmented DNA was processed for final repair, addition of adapters and purified using Agencourt AMPure XP beads (Beckman Coulter Inc., Brea, CA, USA) according to the manufacturer’s instructions. The libraries were amplified by PCR with DNA polymerase (Herculase II) provided by Agilent Technologies and captured with 1 μl biotinylated probes. The hybridization step lasted 16 h at a constant temperature of 60 °C. The captured libraries were then amplified by PCR (16 cycles). After purification with AMPure XP beads (Beckman Coulter Inc., Brea, CA, USA), an enrichment step, identical to the initial capture step, was performed according to the same protocol as described above^26^. Quality control of the final MPXV-enriched libraries was performed using an Agilent 2100 BioAnalyzer (Agilent Technologies, USA). Finally, high-throughput sequencing was performed using a Miseq benchtop sequencer (Illumina) with 300 cycles to generate 2–18 million pairs of 150 nucleotides.

### Genome assembly

The quality of raw reads was initially assessed and filtered using CLC workbench 10.0.1, and the trimmed reads were mapped against all MPXV references extracted from GenBank using the following stringent parameters: 95% of the whole read had to align with at least 95% identity. Mapped reads were then assembled de novo using SPAdes v3.10. To evaluate the accuracy of the assembly, reads were mapped back (CLC workbench 10.0.1, 90% length and 90% identity thresholds) to the resulting contigs. All genomes were annotated using VAPid^27^.

### Phylogenetic analyses

The 10 MPXV genomes specially sequenced in this study were aligned with the 56 MPXV genomes available in NCBI using MAFFT v7.471 (2020/Jul/3)^28^. Cowpox virus (Grisham 1990, X94355) and horsepox virus (Mongolia 1976, DQ792504) sequences were used as outgroups. Both extremities of the preliminary alignment containing missing data, as well as many insertions or deletions (indels), they were trimmed and the length of the final alignment was 204,963 bp. The best-fit nucleotide substitution model, i.e., GTR+G, was selected using the Akaike information criterion (AIC) in jModelTest 2.1.10^29,30^. The Bayesian tree was reconstructed with MrBayes v3.2.7 and the posterior probabilities (PP) were computed with four independent Markov chains for ten million generations with a sampling every 1000 generations and a burn-in of 25%. IQ-TREE^31^ was used to calculate bootstrap proportions (BP) using the maximum likelihood method and 1000 replicates.

### Molecular dating analyses

Molecular estimations of divergence times were carried out on an alignment of the complete MPXV genomes found in the Central Africa clade. Estimations were based on the same methodology as described in “Phylogenetic analyses”. To detect a potential correlation between tip dates and root-to-tip distances, a molecular clock signal was estimated on the ML tree using TempEst v1.5^32^. BEAST v1.10.4^33^ was used for Bayesian MCMC analysis to estimate temporal nodes. As recommended in Patrono et al., we selected the simplest model: strict clock and constant population size with a HKY substitution model and four Gamma categories^7^. An MCMC model was run with 50,000,000 generations and a burn-in of 25,000 sampled trees. All other parameters were by default. BEAST executions were completed checking chain convergence and sufficient sampling of the posterior space (ESS > 200) with Tracer v1.7.1. The final chronogram was generated using TreeAnnotator v1.10.4^33^.

### Coding region analysis

The characterization of the polymorphism in ORFs was carried out based on the genomic annotations of the sequence of the MPXV isolate from Zaire isolated in 1996 (NC_003310). At first, all coding sequences (CDS) were extracted from the reference sequence and used to build a BLAST reference database. We then used BLASTN to compare all the genomic sequences in our Central Africa dataset with our reference database to identify homologs. Homologous genes were grouped by family, and codons aligned using translatorx_vLocal.pl script^34^. All multiple sequence alignments were then edited by Aliview^35^ to analyse synonymous and non-synonymous substitutions and alterations (insertion and/or deletion) in MPXV protein-coding genes.

### Ethical considerations

The investigation of all suspected cases of monkeypox occurring in the CAR was approved by the local ethics committee (CES—*Comité Ethique et Scientifique;* University of Bangui). Moreover, oral and written informed consent was obtained from all included patients. All the experiments carried on mice were done in accordance with relevant guidelines and regulations on the use of laboratory animals including the ARRIVE guidelines. All the experiments were done in accordance with relevant guidelines and regulations. The authorization number UB/FACSS/IPB/CES/20 provided by the CES includes both investigations of human suspected Monkeypox cases and experiments carried on mice.

## Results

### Description of micro-outbreaks occurring in the CAR between 2001 and 2018

Since the first human MPXV case reported in Central Africa in August 1970 in a remote village (Bokenda, DRC)^36^, several confirmed cases have been reported in the CAR. The first micro-outbreak of six cases occurred in a Pygmy camp in January 1984 (four cases confirmed by viral isolation) in Lidjombo (Sangha Mbaéré prefecture) in the extreme southwestern region of the country, where the father of a patient reported hunting a duiker and a monkey that both showed skin lesions^37^. The next four cases were identified in a Bantu family in August 2001 in the city of Bangassou (Mbomou prefecture)^4^. In June 2010, two cases were confirmed in two young Pygmy children who had hunted and eaten a wild rodent in the city of Mongoumba (Lobaye prefecture)^10^. Between December 2015 and February 2016, 10 suspected cases were reported in Bangassou (Mbomou prefecture). The index cases were two brothers in Madigui/Bakouma (Mbomou prefecture)^11^, who also infected their mother and their younger brother. A second cluster was also identified in Bangassou, following nosocomial transmission at the Bangassou health care center from the Madigui/Bakouma index case, with a secondary intrafamilial transmission to the family of the nurse. Another case was reported at the same period in Bakouma (Mbomou prefecture)^11^, but epidemiological investigations suggested that it was not linked to the previous outbreak. In 2017, two new micro-outbreaks were reported. The first was in February 2017 in the city of Bao (Mbomou prefecture) where at least two cases were confirmed, and the second was in April in the city of Mbaïki (Lobaye prefecture) with a single confirmed case^22,23,38^. Finally, between March and April 2018, six cases were detected in the town of Ippy (Ouaka prefecture), followed by three other cases in the town of Rafaï (Mbomou prefecture)^24^. Epidemiological investigations suggested that one of the cases in Rafaï (18c) may be related to the Ippy case (15c). The Ippy case involved a street vendor who visited his girlfriend in Rafaï (18c). Among all the MPXV cases reported in CAR since 1984, we selected 10 isolates from nine micro-outbreaks or isolated cases that occurred between 2001 and 2018 in eight cities, located in four prefectures of the country (Table 1). This selection was based on virological criteria, and in particular the PCR Ct values. We studied thus samples for which the Ct was as low as possible corresponding to a high viral load. The location given here correspond to place of reporting are shown in Fig. 1 and Table 1. The places of exposure are sometimes similar but often difficult to determine.

### Analysis of raw sequencing data

Except for the two libraries from the A1 and A2 samples that were prepared from DNA extracted from neonatal mouse brain biopsies, all other libraries were prepared from DNA extracted from scab or pus samples. Threshold cycle (Ct) values determined using quantitative PCR ranged from 18 to 35 Ct for DNA extracted from new born mouse brain biopsies and scabs, respectively. On average, the enrichment protocol provided Ct values between 15 and 18 for the final libraries. The greatest improvements in Ct values (− 16 Ct on average) were obtained with DNA extracted from primary samples, whereas for DNA extracted from the MPXV isolate, the decrease in Ct values was smaller (− 3 Ct on average) (data not shown). Finally, the ten whole-genome sequences were assembled de novo. Then, the raw reads were mapped back to these draft sequences. On average, 98% of the reads were successfully mapped back to these draft sequences. Moreover, this mapping showed that the proportion of MPXV reads ranged from 50% to more than 90% of the total number of raw reads (Table 2). The average sequencing depth varied from 598× to 8714×.

**Table 2:** Description of raw sequencing, mapping and assembly data.

	A1	A2	A4	A5	A6	B1	B2	015c	018c	038c
Total raw reads	1,994,714	1,665,522	17,123,360	12,105,040	11,959,724	3,984,618	1,752,554	8,213,740	6,128,588	7,594,188
Mapped reads	1,029,173 (51.6%)	1,394,382 (83.7%)	16,601,648 (96.9%)	11,824,012 (98.3%)	11,588,292 (97.7%)	3,822,177 (95.9%)	1,497,765 (85.4%)	3,990,602 (48.6%)	2,907,565 (47.4%)	3,647,374 (48.1%)
Average depth	736.82X	598.32X	8714.24X	6418.99X	6545.91X	1683.79X	620.24X	2613.04X	1867.17X	2309.89X

### Phylogenetic, molecular dating and phylogeographic analyses

**Figure 3:**
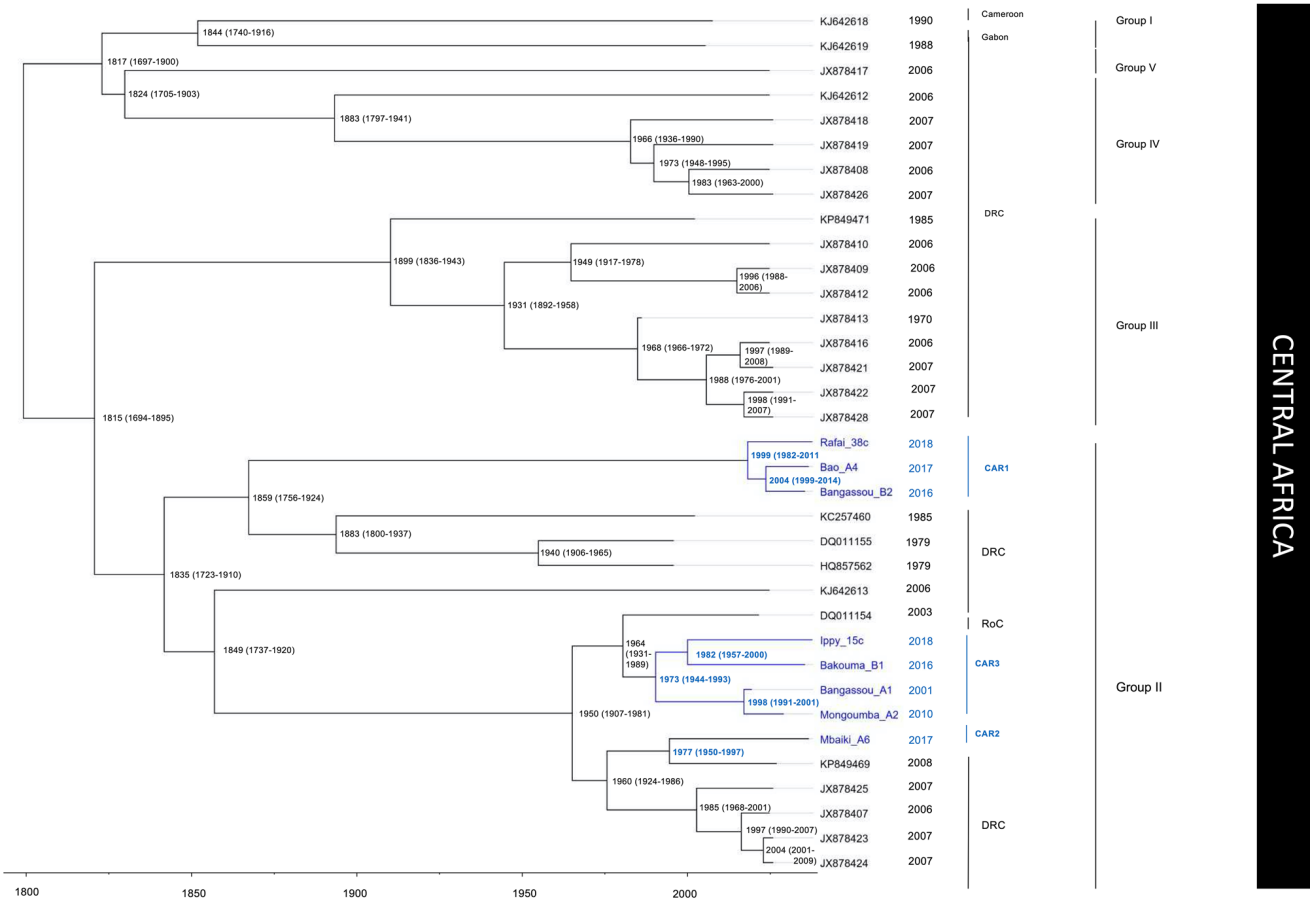
Bayesian chronogram of Central African monkeypox viruses (MPXVs). MPXV ge-nomes sampled between 2001 and 2018 in the Central African Republic are shown in blue. Median divergence times are indicated at the nodes and the 95% highest posterior density (HPD) is given in parentheses.

The Bayesian tree of Fig. 2 shows that the 10 MPXV genomes from the CAR belong exclusively to Group II of the Central Africa clade as defined by Nakazawa et al.^25^. Three different lineages can be distinguished in the CAR. They are hereinafter referred to as CAR1, CAR2 and CAR3. The lineage CAR1 contains four of the six isolates detected in the Mbomou prefecture (B2, A4, A5, and 38c) between 2016 and 2018 (PP = 1; BP = 100). It appears as the sister-group of a cluster of MPXV isolates collected in the DRC in 1979 (Mindembo; DQ011155) and 1985 (Yandongi, a town located a few hundred kilometers south of the CAR border; KC257460), as well as one MPXV found in South-Sudan in 2005 (KC257459) (Fig. 2). Molecular dating estimates suggest that the CAR1 cluster has diverged from its sister-group between 1999 (1982–2011, 95% highest posterior density [HPD]) and 2004 (1991–2014, 95% HPD) (Fig. 3). The lineage CAR2 is represented by a single MPXV collected in the Lobaye prefecture (A6), and it shares a common ancestor with a MPXV detected in the DRC in 2008 (Boende, KP849469) (PP = 1; BP = 94). The lineage CAR3 includes five MPXVs collected in three different CAR prefectures (Lobaye, Ouaka and Mbomou) (PP = 1; BP = 65). It is enclosed into a robust clade (PP = 1; BP = 100) with its sister-group from the DRC, which is composed of five isolates sampled in Lomela and Kole in 2006 and 2007 (PP = 1; BP = 57), the MPXV isolate found in R. Congo in 2003 (DQ011154; Ipfondo), and the CAR2 lineage with its sister-genome from the DRC (Boende). Within the lineage CAR3, the Ippy (15c) sequence isolated in 2018 from the Ouaka prefecture clustered with the sequence isolated from a case in Bakouma (Mbomou) in 2016 (PP = 1; BP = 100), whereas the MPXV found in Bangassou (Mbomou prefecture) in 2010 clustered with the sequence isolated in 2010 in Mongoumba (Lobaye prefecture) (PP = 1; BP = 95). The median divergence time for the common ancestor of CAR3 was around 1973 (1944–1993, 95% HPD). However, the 2001 Bangassou isolate diverged from the Mongoumba 2010 isolate in around 1998 (1991–2001, 95% HPD), only a few years before it was detected in the Bantu family in 2001. This pattern contrasts with the Ippy/Bakouma isolates, which diverged around 1982 (1957–2000, 95% HPD), before their recent detection between 2016 and 2018 (Fig. 3).

### Analysis of coding regions

Using CDS annotations provided by the Zaire-1996 sequence (NC_003310), we extracted, using sequence similarities, 191 homologous CDS for each CAR genomic sequence. These CDS were multiple-aligned to detect variations between the CAR sequences, as well as those belonging to MPXV Congo Basin group II. At least one SNP was detected in 47 different ORFs, even though three of these ORFs were identical because they were located in the inverted terminal regions (ITR) of the genome. In 70.2% (33/47) of the cases, a single SNP was identified in an ORF. However, in 21.3% (10/47), 6.4% (3/47) and 2.1% (1/47) of the other cases, two, three or four SNPs were found in the same ORF, respectively (Table S1). Moreover, 36.4% (24/66) of these SNPs were non-synonymous mutations. All these different SNPs distinguish both the CAR sequences from each other and from other MPXV sequences belonging to group II or the Central Africa clade. However, the number of SNPs specific to each group varied. In fact, 44 SNPs were specific to the CAR1 lineage (38c, A4, A5, and B2) and 22 SNP were specific to CAR2 and CAR3 lineages (15c, 18c, A1, A2, B1 and A6). In addition, there were major differences in the distribution of specific SNPs within each of these subgroups: the B2 genome isolated in Bangassou in 2016 (CAR1) had no specific SNP, but the Bakouma 2016 genome (B1, CAR3) showed eight diagnostic SNPs. Within the CAR1 lineage, the genomes 38c and A4/A5 (which were identical) had only three specific SNPs each. Moreover, the genomes isolated in Bangassou in 2001 (A1) and Mongoumba in 2010 (A2) differed by only one SNP located in the B2R gene (Table S1).

In parallel to the search for these SNPs, a total of 10 alterations, indels of one or more amino acids, were found in nine ORFs. These indels were specific to certain isolates or sequence groups (Table S2). Of the ten detected indels, six are repetitions of a 2- to 9-bases motif. In two identical MPXV genomes (15c and 18c), we found a repetition of a “AT” motif that induces a frameshift generating a truncated B14R protein. These analyses also showed four deletions of 2–19 nucleotides. Two of these deletions result in protein alteration: a frameshift in the B16R gene (15c/18c genomes) or a truncated protein due to a stop codon in the D2L gene of all genomes of the CAR1 lineage (38c, A4, A5 and B2) (Table S2).

## Discussion

This study is the first to describe the genomic data for MPXVs isolated from human cases in the CAR.

First of all, our analysis showed that the 10 CAR MPXV genomes have the typical organization observed in other orthopoxviruses: there is a conserved central region and ITRs with tandem repeats at both extremities. As expected, all ORFs—except four that are located in the ITRs—were present in the central region of the genome. Moreover, the mean genome-wide substitution rate for sequences belonging to the Central Africa clade was estimated at 1.06 × 10^–6^ (5.47 × 10^–7^–1.61 × 10^–6^, 95% HPD) substitutions per site per year. Our estimate is slightly lower than that obtained by Patrono et al. on MPXV sequences belonging to the West Africa clade (1.93 × 10^–6^)^7^ or for the variola virus (8.5 × 10^–6^)^39^. However our estimate remains within the expected range for double-stranded DNA viruses^40^. As expected, the MPXV sequences belonging to the Central Africa clade generally differ from each other by only single nucleotides. There were also some indels, but most of these mutations were located in non-coding regions where tandem repeats have been described previously. There is a single exception, with one insertion in the gene that codes for an interleukin receptor (IL-1β). In fact, this gene has already been identified as one of the less conserved genes between the Central and West Africa clades, which may help to explain the difference in virulence observed between isolates of these two clades^41^. The IL-1β gene produces a protein that prevents cytokine from binding to the IL-1 receptor^42,43^. IL-1, and one of its forms (IL-1β) in particular, is involved in the inflammatory response during infection by binding to IL-1 receptors that activate several signaling pathways^44,45^. In addition, experiments on the vaccinia virus have shown that the IL-1β binding protein inhibits the immune response by affecting the proliferation of murine B and T lymphocytes; mice infected with a deletion mutant are not as ill as those infected with the wild isolate^42,43^. Our analysis shows that several sequences belonging to the Central Africa clade, including the Zaire-1979 and CAR-15c/18c sequences, have an ORF that codes for a truncated protein (210 aa instead of 326 aa in the other isolates). The consequences of this alteration on protein function and the virulence of the virus are not known. Therefore, despite a retrospective analysis of the two CAR cases related to this isolate, we could not conclude as to the potential impact of this alteration on the clinical outcome of the disease. However, patient 18c had more than 180 lesions, which corresponds to a severe clinical outcome^46^. Unfortunately, this rash severity score cannot be compared with the other cases, either in patient 15c or in the nine other patients in this study.

The distribution of MPXVs examined in this study (Fig. 1) shows that all of them were collected in the rainforests of tropical Africa, with the exception of an isolate collected in South Sudan in 2005 and another one detected in CAR in 2018 (Ippy). These two cases were probably imported from rainforest regions. Our tree of MPXVs (Fig. 2) agrees with previous phylogenetic studies showing the existence of five main groups (named I to V) in the clade of Central Africa. The group I contains two isolates collected in the western part of the Congo Basin, i.e., one from Cameroon and another from Gabon. The four other groups were found in the central part of the Congo Basin: the three groups III, IV, and V are endemic to DRC, whereas group II includes isolates from DRC, R. Congo, CAR, and South Sudan (but see below). In addition, the two DRC groups V and IV have separated earlier than other groups (I, II, and III; Fig. 2). Biogeographically, this phylogenetic pattern suggests that MPXVs of the clade of Central Africa have mainly diversified in the rainforests of the DRC. In agreement with that, most small outbreaks detected in CAR can be related to the rainforests of Lobaye and Mbomou (Fig. 1). However, little is known about the wild animals at the origin of human contaminations in the CAR, despite the field investigations that are systematically carried out when a human case is reported (Table 1). Although squirrels have been strongly suspected to be one of the hosts of this virus in Africa, this taxon has not yet been confirmed in any of the investigations carried out in the CAR^47^.

Our data show that many MPXV isolates detected from individual cases or micro-outbreaks identified in the Mbomou prefecture were epidemiologically unrelated, although several of them belong to the same CAR1 lineage, i.e. the isolates from Bao (A4), Rafaï (38c) and Bangassou (B2) collected between 2016 and 2018. Moreover, these isolates might have diverged from each other between 1999 and 2004, whereas the isolates from Bangassou (A1) and Mongoumba (A2) separated around 2001. This period coincides with a period of intense political instability in the CAR, occurring between 1992 and 2005, when a military takeover in 2001 led to a large number of population movements within the CAR or across borders to neighboring countries. These movements associated with increased poverty may have resulted in more frequent contacts with infected animals, perhaps due to increased activities such as hunting or bushmeat trade.

To date, MPXV has been reported in human populations in only four West African countries (Nigeria, Ivory Coast, Liberia and Sierra Leone) and six Central African countries (the RC, the DRC, the CAR, Gabon, Cameroon, and South Sudan)^9^. Over the last two decades, an increasing number of cases have been reported, particularly in the CAR^9^. Improvements in disease surveillance have certainly contributed to such increase in incidence, but not exclusively^48–50^. Other possible explanations include the cessation of routine smallpox vaccination campaigns, which ended between 1978 and 1980. As a result, there was a drop in cross-protection against *Orthopoxvirus* species in these populations^3,51,52^. Other MPXV exposure factors include the generally difficult living conditions, either due to poverty or recurrent wars or civil unrest, which tend to force people to move over long distances, and for some to flee to rainforests. Deforestation has also led to ecosystem changes, leading to local extinction, colonization by invasive species/alien species, and may also foster the enzootic circulation of MPXV^53^. All of these changes may have led to an increase in repeated contact with wild animals potentially infected by MPXV, particularly in children and young male adults^3^. In Nigeria or Sierra Leone, human cases more likely correspond to indigenous zoonotic spillover than to importation from a neighboring country ^14,54^. Although three modes of transmission (familial, health care-related and transport-related) were identified during the small outbreak that occurred in Bangassou in 2016 ^11^, human-to-human transmission in CAR is generally confined to a single family^55^. It rarely spreads over long distances, with the exception of the street vendor from Ippy who probably infected his girlfriend in the town of Rafaï, several hundred kilometers away. Unlike the outbreak in Nigeria where several cases related to it have been reported outside the country, no human case related to a MPXV isolate from the CAR has been reported outside the country to date.

In conclusion, our phylogenetic results indicate that the 10 CAR isolates belong to three lineages closely related to those found in DRC. Furthermore, the phylogenetic pattern shows that all of them emerged in the rainforest block of the Congo Basin. Since most human index cases in CAR occurred at the northern edge of western and eastern rainforests, transmissions from wild animals living in the rainforest is the most probable hypothesis. Ecological disturbances through land-use changes, deforestation and geographical expansion of human activities will probably lead populations to interact more and more with wild forest mammals and thus increase the risk of zoonotic spillover^56^. In addition, molecular dating estimates suggest that periods of intense political instability resulting in population movements within or outside the country often associated also with increased poverty may have led to more frequent contact with host wild animals and to monkeypox human cases. To limit transmission and prevent the spread of MPXV to the human population^8^, new studies must seek to better characterize all CAR isolates that remain unsequenced and all animal hosts still unidentified to date, to evaluate ethological and ecological risk factors involved in human infections.

## Supplementary Information

Supplementary Table S1.

Supplementary Table S2.

## Data Availability

The MPXV nucleotide sequences have been deposited in GenBank under accession numbers MN702444 to MN702453. Raw sequence reads have been deposited in the NCBI SRA database under the BioProject number PRJNA680806.
