# Supplementary material for: Genomic history of human monkey pox infections in the Central African Republic between 2001 and 2018
Source: Sci Rep. 2021 Jun 22;11:13085. doi: 10.1038/s41598-021-92315-8 (PMC8219716; doi:10.1038/s41598-021-92315-8)
Supplement: Supplementary file 1 — Supplementary Table S1. [file 41598_2021_92315_MOESM1_ESM.docx]

**Table S1. Description of the polymorphisms observed in the ORFs of the 10 MPXV sequences from the CAR compared with the other sequences of the Central Africa clade.** The base shown in bold corresponds to the variant base with regard to the other sequences.

| ORF | Size (aa) | Function/feature | Position of SNP | 38c | A4 | A5 | B2 | A6 | 15c/  18c | B1 | A1 | A2 | aa |
| --- | --- | --- | --- | --- | --- | --- | --- | --- | --- | --- | --- | --- | --- |
| A3L | 77 | NA | 116^⊥^ | C | C | C | C | C | C | **T** | C | C | V39A |
| A4L | 644 | Major virion core protein p4b | 1122 | **T** | **T** | **T** | **T** | C | C | C | C | C |  |
| A11L | 891 | Major virion core protein p4a | 807 | **C** | **C** | **C** | **C** | T | T | T | T | T |  |
|  |  |  | 1593 | **T** | **T** | **T** | **T** | C | C | C | C | C |  |
|  |  |  | 2271 | T | T | T | T | T | **C** | T | T | T |  |
| A17L | 377 | Soluble myristylated protein | 492 | G | G | G | G | G | G | **T** | G | G |  |
| A18L | 204 | IMV surface membrane protein, early function in virion morphogenesis | 348 | **A** | **A** | **A** | **A** | G | G | G | G | G |  |
| A21L | 115 | NA | 159 | **C** | **C** | **C** | **C** | T | T | T | T | T |  |
| A23R | 187 | NA | 389^⊥^ | C | **T** | **T** | C | C | C | C | C | C | V130A |
| A27L | 696 | N-terminal of A-type inclusion body protein of CPV | 54 | **C** | **C** | **C** | **C** | A | A | A | A | A |  |
| A30L | 146 | NA | 298^⊥^ | A | A | A | A | A | A | **T** | A | A | S100T |
| A35R | 181 | EEV envelope glycoprotein, needed for formation of actin-containing microvilli and cell-to-cell spread | 228 | **T** | **T** | **T** | **T** | C | C | C | C | C |  |
| A44R | 74 | NA | 85^⊥^ | **A** | **A** | **A** | **A** | G | G | G | G | G | I29V |
| A45L | 346 | 3-B-Hydroxy-delta5-steroid dehydrogenase | 318 | G | G | G | G | **A** | G | G | G | G |  |
| A51R | 334 | NA | 270 | **C** | **C** | **C** | **C** | T | T | T | T | T |  |
| B5R | 561 | Ankyrin-like | 837 | C | C | C | C | C | C | C | C | **T** |  |
| B6R | 317 | Palmitoylated 42 kDa EEV glycoprotein required for efficient cell spread, complement control protein-like | 183 | C | C | C | C | **T** | C | C | C | C |  |
|  |  |  | 369 | **T** | **T** | **T** | **T** | C | C | C | C | C |  |
|  |  |  | 519^⊥^ | **A** | **A** | **A** | **A** | C | C | C | C | C | D173A |
| B10R | 221 | Shope fibroma virus T4 protein-like | 99^⊥^ | A | A | A | A | A | A | A | **C** | **C** | K171R |
| B12R | 344 | Serine protease inhibitor-like, SPI-2, inhibits IL-1B converting enzyme | 652^⊥^ | A | A | A | A | A | A | **T** | A | A | S218T |
|  |  |  | 708 | G | G | G | G | **A** | **A** | G | G | G |  |
| B21R | 1879 | Putative membrane-associated glycoprotein, cadherin-like domain | 377^⊥^ | **G** | C | C | C | C | C | C | C | C | S126T |
|  |  |  | 2708^⊥^ | **G** | **G** | **G** | **G** | A | A | A | A | A | R903K |
|  |  |  | 5522^⊥^ | G | G | G | G | G | G | **A** | G | G | H1841R |
|  |  |  | 5553^⊥^ | A | A | A | A | A | A | **G** | A | A | M1851I |
| C2L | 375 | Serine protease inhibitor-like, SPI-3, prevents cell fusion | 483 | C | C | C | C | **T** | C | C | C | C |  |
| C10L | 319 | Ribonucleotide reductase, small subunit, R2 | 954 | **C** | **C** | **C** | **C** | T | T | T | T | T |  |
| C16L | 439 | Serine/threonine protein kinase 2, VPK2, regulation of virion morphogenesis | 930 | **C** | **C** | **C** | **C** | T | T | T | T | T |  |
| C18L | 635 | Actin tail formation | 821^⊥^ | **A** | **A** | **A** | **A** | G | G | G | G | G | N274S |
|  |  |  | 1805 | **C** | **C** | **C** | **C** | A | A | A | A | A |  |
| C19L | 372 | Major envelope antigen of EEV, wrapping of IMV to form IEV, phospholipase D-like | 570 | C | C | C | C | **T** | C | C | C | C |  |
| D1L* | 437 | Ankyrin-like |  |  |  |  |  |  |  |  |  |  |  |
| D4L | 83 | NA | 109^⊥^ | **T** | **T** | **T** | **T** | G | G | G | G | G | Y37D |
|  |  |  | 213 | C | C | C | C | C | C | **T** | C | C |  |
| D6L | 126 | Secreted IL-18-binding protein | 186^⊥^ | **A** | C | C | C | C | C | C | C | C | E62D |
| D7L | 660 | Host range; ankyrin-like | 269^⊥^ | **C** | **C** | **C** | **C** | T | T | T | T | T | A90V |
|  |  |  | 1326 | **C** | **C** | **C** | **C** | T | T | T | T | T |  |
| D17L | 98 | NA | 110^⊥^ | **T** | **T** | **T** | **T** | C | C | C | C | C | F37S |
| D18L | 107 | NA | 188 | C | **T** | **T** | C | C | C | C | C | C |  |
| E1R | 845 | mRNA capping enzyme large subunit; RNA 5 triphosphatase and RNA guanylyl transferase activities | 1791 | G | G | G | G | **T** | G | G | G | G |  |
| E3R | 233 | Virion core protein | 124^⊥^ | **T** | **T** | **T** | **T** | C | C | C | C | C | Y42H |
| E7R | 161 | RNA pol 18 kDa subunit | 263^⊥^ | **C** | **C** | **C** | **C** | G | G | G | G | G | T88R |
| E8L | 304 | IMV surface membrane 32k Da protein, binds cell surface chondroitin sulfate, IMV adsorption to cell surface | 638^⊥^ | **A** | **A** | **A** | **A** | G | G | G | G | G | H213R |
|  |  |  | 736^⊥^ | **A** | **A** | **A** | **A** | G | G | G | G | G | M246V |
|  |  |  | 864 | C | C | C | C | C | C | **A** | C | C |  |
| F1L | 479 | Poly(A) polymerase, catalytic subunit | 904^⊥^ | **T** | C | C | C | C | C | C | C | C | S302P |
|  |  |  | 1125 | C | C | C | C | C | **T** | **T** | C | C |  |
| F8L | 1006 | DNA polymerase | 1530 | **A** | **A** | **A** | **A** | G | G | G | G | G |  |
| H1L | 171 | Tyrosine/serine protein phosphatase, blocks IFN-gamma signal transduction | 174 | **A** | **A** | **A** | **A** | G | G | G | G | G |  |
| H2R | 189 | NA | 358 | **T** | **T** | **T** | **T** | C | C | C | C | C |  |
| H4L | 795 | RNA polymerase-associated protein, RAP 94, provides specificity for early promoters | 1941 | **G** | **G** | **G** | **G** | A | A | A | A | A |  |
|  |  |  | 2172 | C | **T** | **T** | C | C | C | C | C | C |  |
| I1L | 312 | Virosomal protein essential for virus multiplication | 678 | **A** | **A** | **A** | **A** | G | G | G | G | G |  |
| I4L | 771 | Ribonucleotide reductase, large subunit, R1 | 1035 | **C** | **C** | **C** | **C** | T | T | T | T | T |  |
| I6L | 382 | NA | 330 | **A** | **A** | **A** | **A** | G | G | G | G | G |  |
|  |  |  | 1032 | **C** | **C** | **C** | **C** | T | T | T | T | T |  |
| I8R | 676 | Nucleoside triphosphate phosphohydrolase II, NPH-II, DNA, and RNA helicase | 1050 | A | A | A | A | **T** | A | A | A | A |  |
| J1L*/J3R* | 246 | Secreted CC-chemokine-binding protein | 687 | **C** | **C** | **C** | **C** | T | T | T | T | T |  |
| J1R*/J3L* | 587 | Ankyrin-like | 21 | G | G | G | G | G | G | G | **A** | **A** |  |
|  |  |  | 1297^⊥^ | **C** | **C** | **C** | **C** | A | A | A | A | A | L433I |
| L6R | 1286 | RNA polymerase 147 kDa subunit | 399 | **T** | **T** | **T** | **T** | C | C | C | C | C |  |
|  |  |  | 1497 | **T** | **T** | **T** | **T** | C | C | C | C | C |  |
| N1R | 153 | VAC B15R-like | 393^⊥^ | **G** | **G** | **G** | **G** | A | A | A | A | A | M131I |
| N4R*/D1L* | 437 | CPV C1L, ankyrin-like | 511 | T | T | T | T | **C** | T | T | T | T |  |
| Q1L | 665 | NA | 1116 | **C** | **C** | **C** | **C** | T | T | T | T | T |  |
|  |  |  | 1625^⊥^ | A | A | A | A | A | A | A | **G** | **G** | C542Y |

NA: not available (not known); * : genes located in ITR; ^⊥^:nucleotide sites involved in non-synonymous substitutions.
