# Supplementary material for: Genomic history of human monkey pox infections in the Central African Republic between 2001 and 2018
Source: Sci Rep. 2021 Jun 22;11:13085. doi: 10.1038/s41598-021-92315-8 (PMC8219716; doi:10.1038/s41598-021-92315-8)
Supplement: Supplementary file 2 — Supplementary Table S2. [file 41598_2021_92315_MOESM2_ESM.docx]

**Table S2. Description of the alterations observed in the ORFs for the 10 monkeypox virus (MPXV) sequences of the Central African Republic compared to the other sequences of the Central Africa clade.**

| **Gene** | **Size (aa)** | **Function/feature** | **Alteration** | **number of bases** | **Comments** |
| --- | --- | --- | --- | --- | --- |
| A28L | 520 | Major component of IMV surface tubules, p4c | Insertion | Variable | Variable repeat of the GAT motif. |
| A33R | 142 | NA | Insertion | 9 | Repetition of the TATAACAAT motif. Only found in the 15c/18c sequences. |
|  |  |  | deletion | 9 | AACAATTAT motif, not found in CAR1 sequences (38c, A4/A5 and B1) and in some DRC sequences. |
| A48R | 61 | NA | insertion | Variable | Variable repeat of the TATGAT motif. |
| B14R | 326 | Secreted, IL-1B binding, inhibits virus induced fever | insertion | 4 | Repetition of the TA motif. Induces a frameshift and a truncated protein. |
| B16R | 352 | Cell surface antigen and secreted IFN-a/b- binding protein | deletion | 2 | Deletion of 2 bases at the beginning of the gene. Induces a frameshift. Only found in the 15c/18c sequences. |
| D17L | 98 | NA | insertion | Variable | Insertion of a variable number of A in a homopolymeric region. Only found in Group CAR1 sequences (DRC 79, 85 and 38c). |
| D2L | 64 | VAC C7L-like | deletion | 19 | Frameshift and creation of a truncated protein. Only found in Group CAR1 sequences (38c, A4/A5 and B1). |
| J1L/J3R | 246 | Secreted CC-chemokine-binding protein | deletion | 18 | Found only in sequence 38c from the CAR and sequences from the DRC (1979, 1985 and 2006), Cameroon and Gabon. |
| O1L | 442 | Ankyrin-like | insertion | 3 | “TAT” motif, only found in the 15c/18c sequences. |

NA, not available (not known)
